# Dermatologists might be the first to suspect hereditary leiomyomatosis and renal cell carcinoma syndrome^⋆^

**DOI:** 10.1016/j.abd.2021.09.023

**Published:** 2023-05-23

**Authors:** Elena González-Guerra, Alberto Conde Taboada, José Antonio Cortés Toro, Eduardo López Bran, Pedro Pérez Segura

**Affiliations:** a Department of Dermatology, Hospital Clinico Universitario San Carlos, Madrid, Spain; b Department of Pathological Anatomy, Hospital Clinico Universitario San Carlos, Madrid, Spain; c Department of Genetics, Hospital Clinico Universitario San Carlos, Madrid, Spain

Dear Editor,

Multiple cutaneous and uterine leiomyomatosis (OMIM 150800) is a rare, dominant autosomal hereditary disease in which patients develop multiple cutaneous and uterine leiomyomas. Around 14%‒30% of patients also develop unilateral, solitary, and aggressive renal cell carcinomas (usually type-2 papillary). Consequently, some authors refer to this disease as Hereditary Leiomyomatosis and Renal Cell Carcinoma syndrome (HLRCC).^1^ It is caused by a germline heterozygous mutation of the gene coding for fumarase (1q42-q44), also known as Fumarate Hydratase (FH).^1^ Patients commonly die within 5 years of diagnosis,^2^ so early detection is vital. Since cutaneous leiomyomas are one of the most constant manifestations of this disease, dermatologists might be the first to suspect it; when they do, they should facilitate genetic analysis.

The authors recently examined a 35-year-old woman (with a history of eating disorder (anorexia nervosa) since she was 13, suicide attempts, convulsive crises, irritable bowel syndrome, pollen allergy, and bronchial asthma) who presented with over 20 subcutaneous nodules around her body, some of which were painful, which she had had from adolescence with gradual onset.

Exploration revealed the presence of small, elastic nodules and papules with poorly defined borders, covered by slightly hyperpigmented skin and fibroelastic in consistency (Fig. 1). Some were painful when palpated. Ultrasound examination of the nodules in her left arm and thigh revealed highly vascularised hypoechoic lesions (Fig. 2).

**Figure 1:**
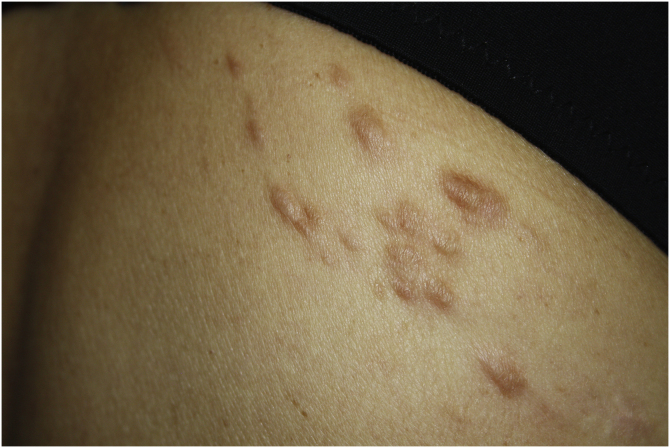
Small and elastic nodules/papules with poorly defined borders, covered by normal skin located on the shoulder

**Figure 2:**
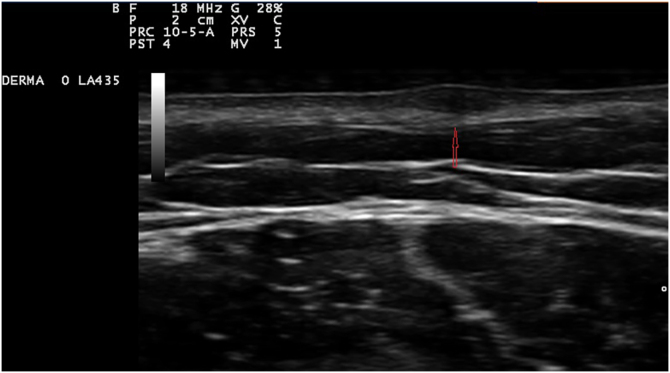
Ultrasonography shows a hypoechoic lesion located in deep dermis (arrow)

Excisional biopsy revealed lesions with a poorly defined border, composed of fascicles of entwined fusiform cells irregularly distributed within the dermis, sparing the superficial dermis (Fig. 3). HLRCC was suspected, and genetic analysis confirmed the patient to carry a p.Arg233Cys mutation in the FH gene.

**Figure 3:**
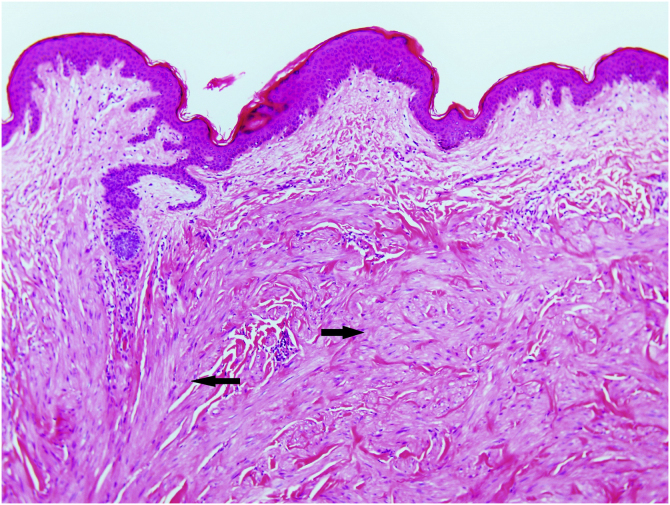
Light microscopy shows a mesenchymal spindle-cell neoplasm located in the dermis, composed of elongated cells of eosinophilic cytoplasm irregularly distributed (arrows). The neoplasm is adjacent to hair follicle (Hematoxylin & eosin, ×40)

The patient was referred for gynecological examination, and multiple uterine leiomyomas were detected. A hysterectomy was performed (despite the patient's age and her having no children), and she was referred for nephrological monitoring, which she continues with no important findings.

Examination of her family members revealed her mother and maternal uncle both have multiple cutaneous leiomyomas as well. Her mother had undergone a hysterectomy for bleeding leiomyomas as a young adult after having two children. Genetic analysis of these relatives revealed the same genetic mutation as the patient; to date neither has developed renal cancer.

HLRCC is characterized by the development of up to three types of tumors: cutaneous piloleiomyomas, which are seen in nearly all patients by the age of 40 years; uterine leiomyomas, which are seen in 70%‒98% of female patients^3^ and usually before the fourth decade of life (mean age at appearance 30 years, range 18‒52 years), and type 2 renal cell papillary carcinoma, which occurs in around 20% of patients (mean age of appearance 46 years, range 17‒75 years).^2^ Cutaneous piloleiomyomas are usually multiple, and appear in both sexes and in more than one area of the body, although more localized distributions have been described in some patients.^4^ Painful piloleiomyomas have been recorded in 90% of patients. This pain could be due to ischemia produced by the contraction of smooth muscle as a reaction to cold, pressure, stress, or an increase in the number of nerve fibers within the lesion.^3^, ^5^, ^6^ Possible transformation to malignant leiomyosarcoma has been described,^6^ although this is controversial.^7^ Diagnosis is confirmed histopathologically via non-encapsulated, poorly defined axes of intertwined fusiform cells seen throughout the thickness of the dermis but leaving a superficial band of the dermis clear. Immunohistochemical staining shows markers of smooth muscle, including actin and desmin.^4^ Llamas et al.^8^ reported that piloleiomyomas associated with HLRCC commonly immunostain weakly or not at all for anti-FH, but strongly for anti-2SC.

Uterine leiomyomas affect 76%‒100% of women with a mutated gene for FH.^4^ Bleeding may require a hysterectomy at an early age.^4^ Their transformation into very aggressive leiomyosarcomas has been described.^9^

Around 20% of patients with HLRCC may also develop type 2 papillary renal cell carcinoma^2^ (the most common renal carcinoma in such patients). Its appearance is generally unilateral (unlike that seen in other hereditary renal cancer syndromes such as Von Hippel Lindau or Birt-Hogg-Dubé syndrome) and tends to be aggressive.^1^ No efficient therapy exists;^2^ patients should undergo yearly check-ups and small renal tumors should be surgically removed.^2^, ^10^

The FH enzyme takes part in the Krebs cycle, promoting the transformation of fumarate into L-malate. It has been proposed that the inactivation of FH causes the accumulation of fumarate in cells to about 200 times its normal concentration.^11^ This leads to modifications in the levels of functional Vascular Endothelial Growth Factor (VEGF), erythropoietin, and glucose transporter 1, which together promote the growth of microvessels and the transcription of genes involved in cell survival and proliferation.^11^ The p.Arg233Cys mutation of the FH gene is described in the LOVD database (https://databases.lovd.nl/shared/genes/FH) and has been associated with a 47% loss in FH enzyme activity. Not all patients show the same phenotype, however, which suggests the involvement of additional genetic modifiers or environmental factors in the development of HLRCC.^9^ Germline mutations of the FH gene have also been associated with Leydig cell tumors, mucinous cystadenoma of the ovary, cerebral cavernoma,^11^ a number of suprarenal manifestations,^2^ type 1 endocrine neoplasia syndrome, rheumatoid arthritis, breast, prostate and bladder cancer, and renal and ovarian cysts. ^3^

Given the range of conditions associated with HLRCC, a set of diagnostic criteria has been established (Table 1),^2^ with the presence of multiple cutaneous leiomyomas deemed to be sufficient cause to request genetic analysis. Patients may first seek medical consultation because of these skin lesions, leaving the dermatologist well-positioned to detect this hereditary disease.

**Table 1:** Diagnostic criteria for HLRCC.^2^ To confirm a diagnosis, the major criterion or two or more minor criteria must be met

Diagnostic criteria for HLRCC syndrome
Major criterion
Multiple cutaneous piloleiomyomas confirmed by biopsy
Minor criteria
Surgical treatment for symptomatic leiomyomas before reaching 40 years of age. While uterine leiomyomas are quite common in the general population, patients with HLRCC may require surgery before reaching 30 years of age.
Type-2 renal cell carcinoma before reaching 40 years of age.
Having a first-degree family member with one of the above criteria.

## Financial support

None declared.

## Authors’ contributions

Elena González-Guerra: Approval of the final version of the manuscript; critical literature review; data collection, analysis and interpretation; effective participation in research orientation; intellectual participation in propaedeutic and/or therapeutic; management of studied cases; manuscript critical review; preparation and writing of the manuscript; statistical analysis; study conception and planning.

Alberto Conde-Taboada: Approval of the final version of the manuscript; critical literature review; data collection, analysis and interpretation; effective participation in research orientation; intellectual participation in propaedeutic and/or therapeutic; management of studied cases; manuscript critical review; preparation and writing of the manuscript; statistical analysis; study conception and planning.

José Antonio Cortés Toro: Approval of the final version of the manuscript; critical literature review; data collection, analysis and interpretation; effective participation in research orientation; intellectual participation in propaedeutic and/or therapeutic; management of studied cases; manuscript critical review; preparation and writing of the manuscript; statistical analysis; study conception and planning.

Eduardo López-Bran: Approval of the final version of the manuscript; critical literature review; data collection, analysis and interpretation; effective participation in research orientation; intellectual participation in propaedeutic and/or therapeutic; management of studied cases; manuscript critical review; preparation and writing of the manuscript; statistical analysis; study conception and planning.

Pedro Pérez-Segura: Approval of the final version of the manuscript; critical literature review; data collection, analysis, and interpretation; effective participation in research orientation; intellectual participation in propaedeutic and/or therapeutic; management of studied cases; manuscript critical review; preparation and writing of the manuscript; statistical analysis; study conception and planning.

## Conflicts of interest

None declared.
